# Social Inequalities Along the Childhood Cancer Continuum: An Overview of Evidence and a Conceptual Framework to Identify Underlying Mechanisms and Pathways

**DOI:** 10.3389/fpubh.2019.00084

**Published:** 2019-04-18

**Authors:** Friederike Erdmann, Maria Feychting, Hanna Mogensen, Kjeld Schmiegelow, Hajo Zeeb

**Affiliations:** ^1^Section of Environment and Radiation, International Agency for Research on Cancer (IARC), Lyon, France; ^2^Department of Prevention and Evaluation, Leibniz - Institute for Prevention Research and Epidemiology - BIPS GmbH, Bremen, Germany; ^3^Childhood Cancer Research Group, Danish Cancer Society Research Center, Copenhagen, Denmark; ^4^Unit of Epidemiology, Institute of Environmental Medicine, Karolinska Institutet, Stockholm, Sweden; ^5^Department of Pediatrics and Adolescent Medicine, University Hospital Rigshospitalet, Copenhagen, Denmark; ^6^Health Sciences Bremen, University of Bremen, Bremen, Germany

**Keywords:** childhood cancer, social inequalities, conceptual framework, pathways and underlying mechanisms, social determinants, incidence, survival, survivors

## Abstract

Inequalities in health according to social conditions are regarded as unnecessary and unjust. There is a large body of evidence on inequalities in adult cancer, observable throughout the societies on a national level as well as on a global scale. Socioeconomic influences on health matter at all ages including childhood, for which childhood cancer is the leading cause of disease related death in high-income countries (HICs). Substantial differences in the reported incidence of childhood cancers have been observed globally by socioeconomic development of a population. This is reflected in the higher incidence rates reported for HICs, particularly for acute lymphoblastic leukemia, and for cancer in infants (below 1 year), compared to low- and middle-income countries (LMICs). Considerable inequalities between populations and degree of socioeconomic development are also noted for survival from childhood cancer, with substantially lower survival rates seen in most LMICs compared to HICs. With respect to inequalities by socioeconomic position (SEP) within countries, findings of an association between SEP and childhood cancer risk are diverse and limited to studies from HICs. On the contrary, observations on social inequalities in survival within countries are accumulating and indicate that survival inequalities do not only concern resource-poor countries but also high-income populations including European countries. In turn, a childhood cancer diagnosis in itself may have implications on the parents' socioeconomic situation as well as on the later socioeconomic life after having survived the disease. The underlying mechanisms and causal pathways of these empirically demonstrated social inequalities are poorly understood, although it is of significant public health relevance for any actions or strategies to reduce childhood cancer-related inequity. We propose a conceptual framework on potential underlying mechanism and pathways specifically addressing social inequalities in childhood cancer and after childhood cancer to (i) illustrate potential pathways by which social determinants may create health inequities at different points of the childhood cancer continuum; (ii) illustrate potential pathways by which a childhood cancer diagnosis may impact the socioeconomic situation of the concerned family or the later life of a childhood survivor; and (iii) point out how major determinants may relate to each other.

## Introduction

There is a large body of evidence on inequalities in health including non-communicable diseases such as cancer, indicating that social inequalities affect cancer incidence, survival and mortality on a regional, national, and global level (1–5). Patterns of striking social inequalities in cancer incidence and survival are observable throughout the societies on a national level as well as on a global scale between countries differentiated by level of socioeconomic development (1, 6, 7). The World Health Organization's (WHO) Commission on Social Determinants of Health understands health inequities as inequalities in health that are socially produced, systematic in their distribution across the population or between populations, and unnecessary and unjust (8) and subsequently stated: “Social injustice is killing people on a grand scale” (9).

Socioeconomic influences on health matter at all ages including childhood, for which childhood cancer is one of the most dreaded diseases and the leading cause of disease-related deaths among children 1–15 years in high-income countries (HICs) (10). The Commission on Social Determinants of Health calls for global action on the social determinants of health to reduce health inequity between and within countries and stresses the importance to put major emphasis on early child development and promote health equity from the start of life (9). An essential basis for any action or strategies to reduce cancer-related health inequity is a profound understanding of the underlying mechanisms leading to these social inequalities. While mechanisms and pathways of social inequalities in cancer have been extensively studied in adults, this does not hold true for childhood cancer. Childhood cancer is a heterogeneous group of malignancies with different patterns of etiology (11), incidence (12), anticancer therapy, supportive care, survival rates (13) and late effects (14, 15), and it is therefore likely that patterns of social inequalities as well as their underlying mechanisms not only differ from those in adult cancer but also vary between types of childhood cancer.

The overall aim of this paper was to deliberate on the underlying mechanisms and pathways of social inequalities in relation to childhood cancer, understanding social inequalities in childhood cancer in a most comprehensive way by considering the entire course of childhood cancer from occurrence, diagnosis, morbidity, survival, and consequences for affected patients and their families, treated as a continuum—“the childhood cancer continuum,” and taking a global perspective including inequalities within societies on a national level as well as between countries on a global scale. At first we introduce into the matter by giving a narrative and critical overview of the current empirical knowledge on social inequalities in childhood cancer. Subsequently, we propose a conceptual framework of potential mechanisms and pathways of social inequity along the childhood cancer continuum linking social determinants with (health) inequity along the childhood cancer continuum and illustrate pathways of socioeconomic consequences for childhood cancer patients and their families, with the ultimate aim to help directing scientific research and identify potential targets for future interventions and policy strategies to reduce childhood cancer-related inequity.

When summarizing the current empirical observations on social inequalities in childhood cancer, the unprejudiced term “inequalities” is used. However, following the Commission on Social Determinants of Health (8) we use the judgmental terms equity and inequity for the presentation of our conceptual model as we regard the social inequalities in childhood cancer as unfair and to highlight the normative dimension of empirically demonstrated inequalities (16).

## Overview of Empirical Observations on Social Inequalities in Childhood Cancer

### Inequalities in Childhood Cancer Between Countries

#### Patterns of Childhood Cancer Incidence

**Figure 1:**
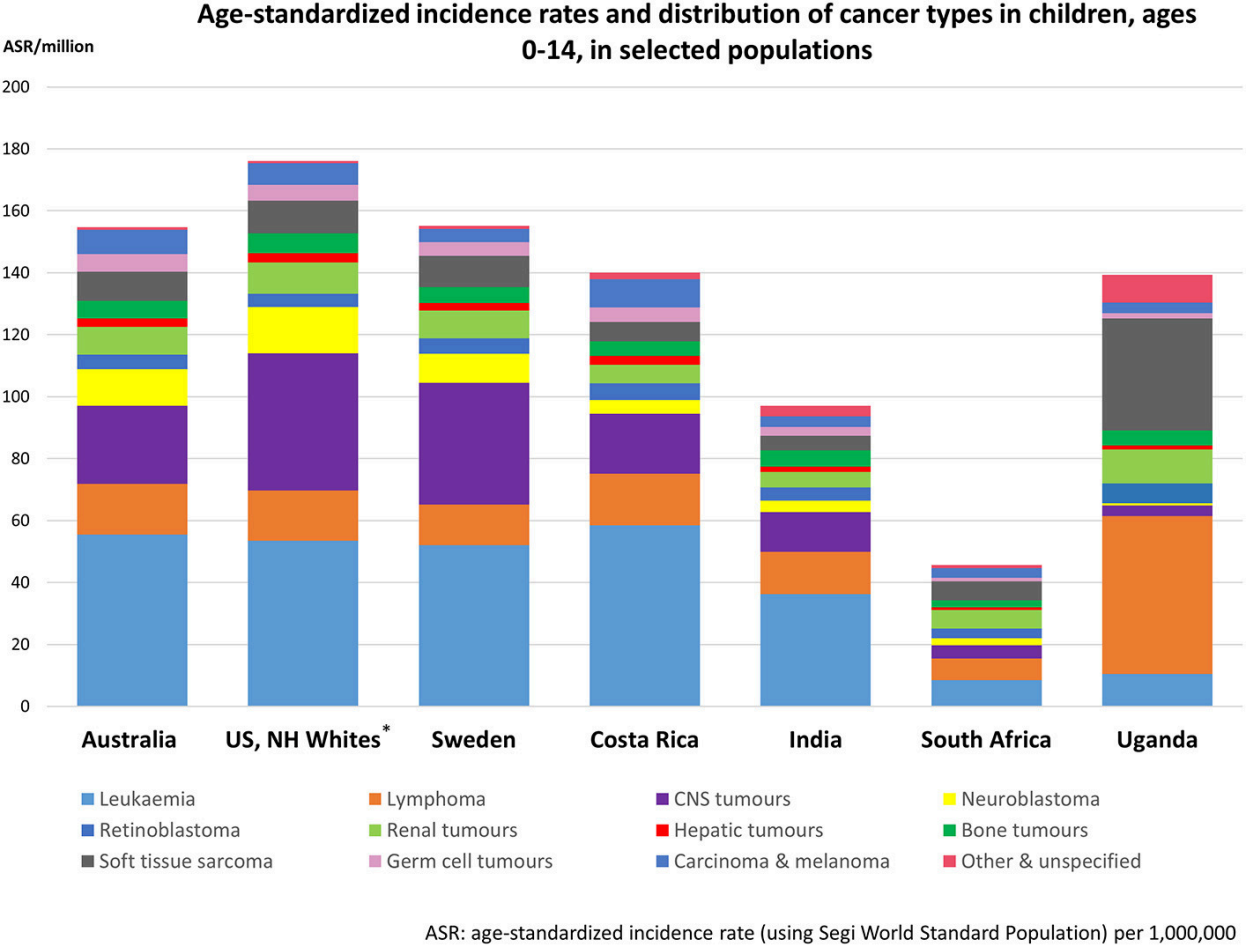
Observed age-standardized incidence rates and distribution of cancer types in children, ages 0–14 in selected populations, ordered by degree of socioeconomic development. Data compiled from the International Incidence of Childhood Cancer, Volume III (12) and cancer register data from Costa Rica (25) and South Africa (26). Diagnostic groups defined according to the International Classification of Childhood Cancer, including non-malignant intracranial and intraspinal tumors. Exceptions are the CNS tumor rates of Costa Rica, India, South Africa, and Uganda; these rates do not include non-malignant brain tumors. ^*^Non-Hispanic Whites.

Population-based cancer registries around the world report overall incidence rates for childhood cancer (cancer in children aged 0–14 years) that vary by a factor of about four, between less than 60 to more than 200 per million per year (12, 17). The socioeconomic development of a population seems to be associated with the reported incidence of childhood cancer in the respective country (18, 19). This is reflected in the higher incidence rates reported for HICs, particularly for acute lymphoblastic leukemia, the most common cancer type in children in HICs (12), and for cancer in infants (below 1 year), compared to low- and middle-income countries (LMICs) (12, 18, 20–24). Childhood cancer incidence patterns are similar and well-described for high-resource countries (12), with recent age-standardized incidence rates of 155, 176, and 155 per million children being reported for Australia (12), US Non-Hispanic Whites (12), and Sweden (12), respectively. In contrast, high-quality data from LMICs is limited and reported incidence patterns are diverse. Incidence rates of 140, 97, 46, and 139 per million have been reported for Costa Rica (25), India (12) South Africa (26), and Kampala in Uganda (12), with substantial variations in the distribution of cancer types among LMICs and in comparison to HICs (12) (Figure 1). For example, in Sub-Saharan Africa, Burkitt lymphoma, Hodgkin lymphoma, Kaposi sarcoma, or hepatocellular carcinoma are more frequent (12, 17). On the other hand, in some LMICs, particularly in Sub-Saharan Africa and parts of Asia where registry data is poor, remarkably low childhood leukemia rates have been observed (17). In contrast, incidence rates of acute lymphoblastic leukaemia (ALL) for some Latin-American countries rank amongst the highest in the world, while lower incidence rates compared to HICs are observed for most solid tumors including malignant central nervous system (CNS) tumors (12, 25). However, estimating and comparing the incidence of childhood cancer globally is impeded by a lack of reliable data for a substantial part of LMICs (26–28).

#### Patterns of Childhood Cancer Survival

Over the past decades, advances in molecular biology, imaging, and chemotherapy with treatment stratification directed by the somatic mutations and early response to chemotherapy, better use of conventional anticancer agents, and improved supportive care, have led to considerable improvements in cure rates of childhood cancers (13, 29–31). In HICs, the 5 year survival of childhood cancer overall has improved from 30% in the 1960s to more than 80% nowadays (13, 32, 34).

**Figure 2:**
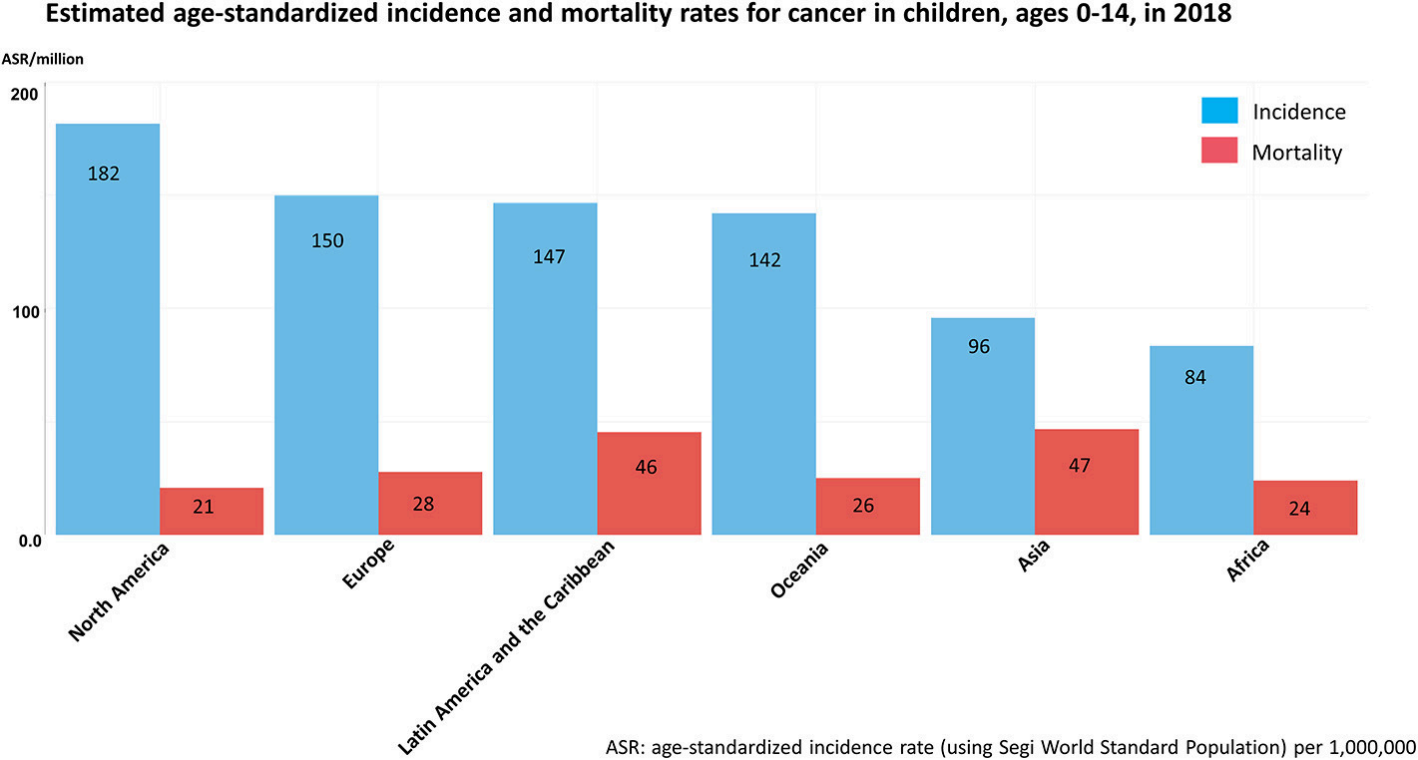
Estimated age-standardized incidence and mortality rates for cancer in children, ages 0–14 in 2018 based on GLOBOCAN 2018 estimates of cancer incidence and mortality. Figure compiled from Global Cancer Observatory (38).

However, not all children benefit from these improvements and substantial differences in survival rates are seen between countries. These inequalities are observed within both high-resource regions such as Europe, with ~10% poorer survival in Eastern European countries compared to the rest of Europe (13), and to a much larger extent in LMICs (34, 35). Similarly to the incidence of childhood cancer, reliable data on childhood cancer survival in LMICs is scarce, but suggests considerably lower survival rates (22, 23, 36, 37) than those observed in HICs (13, 32–34). Mortality-to-incidence ratios give some indications of survival rates (Figure 2). Contrary to the reported incidence, cancer mortality is much higher in resource-poor regions compared to HICs. For instance, for 2018, childhood cancer mortality in Asia was estimated as 47 per million children which represents about 49% of the estimated incidence, while in North America the mortality/incidence ratio is <13% (estimated mortality of 21 per million children) (38).

### Inequalities in Childhood Cancer Within Countries

#### Childhood Cancer Risk

The etiology of most childhood cancers is still poorly understood. The early age at diagnosis suggests that some childhood cancers might originate *in utero*, and that factors prior to birth, including pre-conceptional or fetal environmental exposures, as well as those in early childhood may be important risk determinants (39, 40). A growing body of recent research is directed toward parental exposure to known environmental carcinogens as possible risk factors but have hitherto provided inconsistent results (11, 41). Increasing attention is also given to differences in risk between socioeconomic groups.

Evidence for the association between parental socioeconomic position (SEP) and childhood cancer risk derives solely from HICs and does similarly not provide a consistent picture (42–50). The relationship with socioeconomic factors has been most exhaustively studied for leukemia risk (42–50). Two reviews, summarizing the evidence on socioeconomic factors and leukemia risk (48, 51), interpreted the evidence as heterogeneous with negative associations, positive associations and no associations being observed and studies varying by design, time period, place, leukemia type and measures of SEP used (individual, family, ecological). Recently published findings from Norway showed an association between lower family income and lymphoid leukemia, while a reverse association was reported for myeloid leukemia (50). In Minnesota/USA neither maternal education nor a neighborhood SEP indicator were strongly associated with childhood cancer including leukemia (52) and similar results were seen in Switzerland (49).

Little is known about associations with SEP and the risk of other types of childhood cancer. A large pooled study from the US found an indication of an association between lower parental education and a higher risk of both Hodgkin and Burkitt lymphomas and for Wilms tumor. In contrast, a possibly protective association of lower parental education with astrocytoma and hepatoblastoma was demonstrated (44). Conversely, the Norwegian study (50) found a 70% increased risk for astrocytomas in the medium income category of the parents compared to parents with high income. However, this finding was based on small numbers.

#### Survival From Childhood Cancer

The evidence on social inequalities in survival in LMICs is sparse, available data limited and studies are largely regional within individual countries (53–57), but low SEP was uniformly associated with inferior survival (56).

In HICs, a large number of studies has been examining the association between parental SEP and survival from childhood cancer, particularly during recent years (58). Notably, socioeconomic inequalities in survival have been even found in HICs in which children and adolescents have free and (presumed) equal access to health care services, irrespective of their SEP (58–65). A study in England, Scotland, and Wales observed markedly higher ALL survival among more affluent socioeconomic groups, measured by both area-based deprivation scores and father's occupational status (61, 62). A study from Ireland observed weak trends in survival from ALL in relation to SEP, but no clear evidence was found for other childhood cancer types (66). Findings from Greece indicated an association between parental socio-professional level and ALL, with 40% worse survival for the offspring of parents with lower socio-professional level (60). In contrast, a study from West Germany found no differences in survival from childhood ALL in relation to parental education or family income (67), similar to findings for leukemia in a study from Switzerland (65). The Swiss study observed, however, strong evidence of survival differences among children with CNS tumors, with 50% worse survival in children from less educated families and 30% worse survival in children of the lowest SEP group of the area-based index (65).

In the Nordic countries, population-based nationwide register studies (including all childhood cancer types) from Norway, Sweden and Finland observed a reduced mortality for childhood cancer cases with highly educated mothers (59, 63, 68). In Norway, differences were most pronounced for tumors requiring longer treatment (59), whereas in the Swedish and Finnish studies, survival differences linked to maternal education were indicated for both leukemia (ALL and lymphoblastic lymphoma in the Finnish study) and CNS tumors (63, 68), although not statistically significant. In contrast, among Danish children higher maternal education was only associated with better survival in children diagnosed with a non-CNS solid tumor (64, 69). An association between lower income and higher mortality was observed in Finland. This association was at best suggestive for Denmark, but not found in Sweden and Norway (59, 63, 64, 68). Overall, the specific associations with socioeconomic factors for specific tumor types are inconsistent and contradictory within Europe and within the Nordic countries—a setting with similar welfare systems, longstanding, largely standardized diagnostic, and treatment procedures for childhood cancers and close collaboration between pediatric oncologists.

Considering the evidence from outside Europe, findings from California, a US state with no universal access to health care, neighborhood SEP was not associated with survival from childhood leukemia; however, survival rates were lower for children with no health insurance or an unknown status of insurance coverage (70).

Altogether, although survival inequalities exist in HICs, most children with low SEP will still do better than the children with cancer with higher SEP in resource-poor settings.

### Implications for the Socioeconomic Situation of Parents and Care Givers

Observations from European countries, North America, and other HICs indicate that having a child with cancer may considerably affect the parents' socioeconomic situation. Work disruptions such as time off work, reducing or entirely leaving paid employment and corresponding income deteriorations are highly prevalent among parents of a child with cancer, particularly concerning mothers (71–77) and occurring shortly after diagnosis during the child's active treatment period (72, 76, 78). A study in the US observed that 15% of the families of a child with advanced cancer fell below the poverty line due to this specific situation (79). However, the long-term implications and temporal patterns of such adverse implications on the parental socioeconomic situation are largely unknown and may strongly depend on welfare system and social support in the respective country. The few studies today report conflicting findings regarding the long-term socioeconomic implications (73, 74, 77, 80). Apart from work- or income-related disruptions, parents also reported substantial medical and non-medical expenses that additionally contribute to the parents' and families' economic consequences (81).

Although the scientific knowledge for LMICs is sparse and depending on the welfare system including access to and organization (including funding) of health care services in the respective setting, the socioeconomic implications of childhood cancer for the parents and family are assumed to be substantial in recourse-poor setting (82–84).

### Inequalities in Childhood Cancer Survivors

As a result of improving survival rates, the number of childhood cancer survivors increases continuously. According to predictions for the United States the prevalence of childhood cancer survivors is supposed to approach 500,000 by 2020 for this country alone (85). Whereas, somatic late effects attributable to cancer or its treatment have been addressed in numerous studies (86–89), less is known about the socioeconomic conditions in long-term childhood cancer survivors. A recent, wide-ranging systematic review points out that childhood cancer survivors are at increased risk of adverse socioeconomic outcomes with respect to attendance of special education or learning disability programs, school performance, highest attained education, income level and uptake of social security benefits (90). Several population-based studies observed a lower educational attainment in childhood cancer survivors compared to the general population (91–93), although findings from Switzerland rather suggest a delay in educational achievement than a permanent difference (94).

The evidence on employment status and occupational class in childhood cancer survivors is not as consistent. Two systematic reviews and meta-analyses particularly focusing on work life and employment situation revealed that survivors were 1.5–2 times more likely to be unemployed than people who did not suffer from cancer during their childhood (95, 96). Mader et al. observed considerable differences across regions and cancer types; particularly survivors from the US and Canada and survivors of CNS tumors were more likely to be unemployed (96). Looking at the individual studies, findings from the US indicated that survivors of childhood cancer were at increased risk for unemployment or more likely to have lower-skilled occupations compared to their siblings or the general population (97–100). However, findings from Europe are less conclusive with some reporting higher unemployment rates among European survivors compared to the general population (101–103), whereas others did not observe increased unemployment rates (104–108).

Both unemployment and lower educational attainments have a substantial impact on the survivors' financial situation, and survivors' income has been shown to be markedly lower compared to their siblings or the general population (101, 108, 109).

Notably, survivors of CNS tumors, survivors treated with cranial radiotherapy and those diagnosed at younger age irrespective of cancer type, seem to be at particular risk of adverse socioeconomic outcomes (90).

## Discussion on Underlying Mechanisms and Pathways of Social Inequalities in Childhood Cancer

The underlying mechanisms and causal pathways of these empirically demonstrated social inequalities along the childhood cancer continuum are poorly understood. These mechanisms are likely to differ from those for cancer in adults. For instance, the well-documented relationship between SEP and cancer survival in adults (2) is associated with differences in the time of diagnosis, biological characteristics of the tumor, treatments given and individual characteristics, such as lifestyle or the presence of co-morbidities (3, 5, 110). However, social inequalities in survival from childhood cancer would, at least in most high-resource settings, not be expected to be related to inequalities in co-morbidities, children's lifestyle or treatment (as children have free and equal access to health care services). Moreover, the implications of a cancer diagnosis for later socioeconomic achievements, for instance educational or occupational attainments, in survivors are likely to differ substantially between childhood as compared to adult cancer survivors, where a certain educational level and occupational position has been reached by many patients prior to cancer occurrence.

Understanding the mechanism and pathways leading to these social inequities as well as understanding how a childhood cancer diagnosis affects the socioeconomic situation of the family or the later life of survivors is essential for actions and strategies to tackling inequities.

Theoretical frameworks are helpful to promote understanding and direct investigations on the social determinants of health and health inequalities. There are many frameworks illustrating how those determinants may operate and how they can be improved to reduce social inequities in health (8, 111). However, to our knowledge none of the existing frameworks is particularly suited to elucidate underlying mechanisms and pathways of social inequities in childhood cancer.

## Conceptual Model

**Figure 3:**
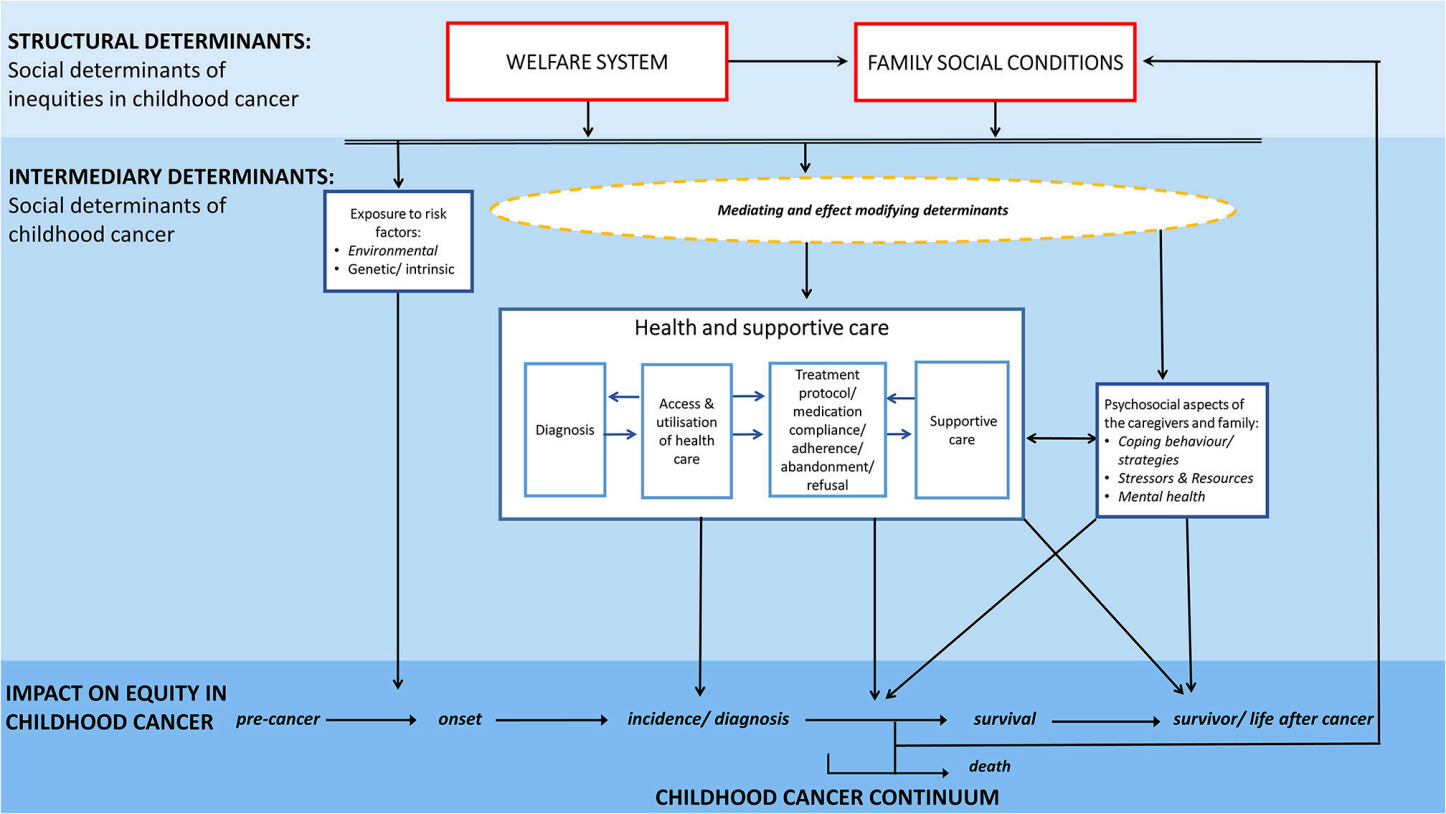
Conceptual model illustrating potential mechanisms and pathways of social inequities along the childhood cancer continuum.

On the basis of the Commission on Social Determinants of Health framework (8) we developed a conceptual model (Figure 3) specifically addressing underlying mechanisms and pathways of social inequities in childhood cancer and after childhood cancer. The conceptual model aims to (i) illustrate potential pathways by which social determinants may create health inequities at different points of the childhood cancer continuum; (ii) illustrate potential pathways by which a childhood cancer diagnosis may impact the socioeconomic situation of the concerned family or the later life of a childhood cancer survivor; and (iii) point out how major determinants relate to each other.

Our framework clearly distinguishes between social determinants of childhood cancer—‘*the social cause impacting on the childhood cancer continuum*' vs. social determinants of inequities in childhood cancer—‘*the social conditions determining the distribution of this social causes across social groups'* (Figure 3). It thereby distinguishes between the mechanisms by which social inequities are created and the conditions of life which then result and impact directly on health and well-being: this is the childhood cancer continuum in our scenario (8). We consider the social determinants of inequities in childhood cancer as *structural determinants* and the social determinants of childhood cancer as *intermediary determinants* (Figure 3).

### Social Determinants of Inequities in Childhood Cancer

In this conceptual model family social conditions are understood in a broad sense, not solely encompassing traditional characteristics such as education, income, and occupation but also extending to health literacy, living and working conditions, social networks, and resources as well as cultural background.

### The Childhood Cancer Continuum

Childhood cancer and related social determinants are seen from a temporal perspective along the course of disease. The childhood cancer continuum is the horizontal axis for this framework and depicts the course of childhood cancer—from disease-free period through pre-clinical phase to diagnosis, established morbidity, survivorship, and to the end of life.

### Pathways of Social Inequities

Various different underlying mechanisms and pathways including several external, intrinsic and cancer-related determinants and a complex interplay of those are likely to contribute to the empirically observed social inequities along the childhood cancer continuum. Key pathways of social inequities may travel from family social conditions through determinants related to health and supportive care including access and utilization of health care services, diagnosis, treatment, supportive care as well as psychosocial aspects related to the cancer diagnosis and its management. Moreover, important associations might also exist with determinants unrelated to health care, e.g., exposures to risk factors or the psychosocial constitution and resources of the concerned family. Pathways related to health and supportive care as well as psychosocial aspects are solely affecting inequities in morbidity, survival and survivorship, while social differences in the exposure to risk factors affect the development of childhood cancers across social groups.

As the drivers that influence childhood cancer occurrence are different from those of inequities in survival and survivorship, so are the underlying mechanisms and pathways they take likely to differ. Similarly, the underlying mechanisms and pathways are likely to vary substantially between types of childhood cancer and related treatment duration and intensity, side effects, psycho-social and socioeconomic impact on the family, as well as late effects.

#### Incidence and Risk

The observed differences in the occurrence of some childhood cancer types by socioeconomic group within a HIC are likely to reflect differences in exposure to some environmental pollutants or (parental) behavioral, biological, or genetic risk factors (41) that vary by socioeconomic groups. This mechanism should apply equally to HICs and LMICs, but how prevalent the exposure is in each socioeconomic group may vary between settings. Moreover, the social context is obviously of relevance for the probability of getting diagnosed: some countries, particularly in resource-poor settings, have large differences in access to, utilization of and quality of health care services by SEP which is likely to have resulted in under-diagnosis of cases (18, 23).

The underlying reasons for the observed geographical differences in incidence rates between countries across the world are not well-known. Differences in genetic or environmental exposures that affect the risk of childhood cancers or certain types may play a limited role. The high incidence rates of Burkitt lymphoma, Hodgkin lymphoma and Kaposi sarcoma in Sub-Saharan Africa (shown in Figure 1 for Uganda) are related to the specific exposure to infectious diseases in that region (namely Epstein-Barr virus, malaria, HIV, and human herpes virus 8) (18, 112, 113). However, for some other cancer types several recent reports indicate that under-diagnosis and under-reporting of cancer cases, at least of leukemia and CNS tumors, may be sufficiently large to account for the majority of the observed differences between some LMICs compared with Europe and North America (17, 26, 28, 114). The empirically reported differences may thus ultimately be related to inequities in access, utilization and quality of health care services including “missed diagnosis” (22, 24, 25), high prevalence of other infectious diseases (e.g., malaria, tuberculosis, HIV/AIDS), high proportions of deaths from unknown causes and differences in (childhood) cancer reporting standards (115). Registries in LMICs are often facing substantial challenges with respect to reporting information on cases and linkage between cancer registries (if they exist); appropriate diagnostic and health care facilities are not always in place or available only at central level (18, 19, 116).

#### Diagnosis, Treatment, Supportive Care, and Survival

Underlying mechanisms and pathways of social inequities in survival may be particularly complex and are likely to involve multiple and potentially interacting determinants.

The large survival inequities observed globally between countries by socioeconomic development are likely predominantly related to limited access to (or utilization of) health care services including contemporary therapy in poor resource settings. Access and utilization are again related to various determinants including poor financial resources, poor organization of health care, poor public transportation infrastructure, cultural beliefs (e.g., belief in traditional medicine) as well as importantly limited access to first-line diagnostics leading to lack or incorrect diagnosis and therapy. An unknown but high proportion of children in LMICs with potentially curable cancer never receives contemporary therapy or, may even not be able to access basic health care services provided by a trained oncologist (34, 117, 118). Primary healthcare facilities and local/regional hospitals may lack awareness of and experience in diagnosing pediatric cancer (18, 19, 114) and the non-specific nature of many early symptoms for some cancer types (e.g., leukemia which often presents with symptoms similar to those of infections) may result in delayed diagnosis or failure to detect the disease at all (34, 115). Even if a childhood cancer is diagnosed, childhood cancers are complex diseases and risk group adapted therapy is crucial (34, 119). Lack of access to first-line diagnostics including pathology services, genetics and high resolution imaging negatively influences outcomes (34).

Even when symptom clearly indicate that a child is suffering from cancer, culture attitudes toward concealment of a cancer in the family, nihilistic beliefs about its curability and trust in traditional medicine may prevent or subsequently delay diagnosis and contemporary therapy (18). Traditional medicine and cultural beliefs continue to play an important role in healthcare delivery in parts of Sub-Saharan Africa, particular among the Black population (118, 120).

Moreover, malnutrition, frequent treatment abandonment or refusal of treatment, co-morbid infections (e.g., HIV) toxic deaths and avoidable relapse are important contributing factors (18, 19, 34, 117, 121, 122) to poor survival in LMICs.

Pathways of survival inequities within countries are likely to vary substantially between countries and regions (such as between high-resource and resource-poor countries) and type of cancer. The pathways outlined in the previous paragraphs concern mainly families with lower SEP in LMICs and apply also to the observed inequalities with respect to low SEP inferior survival in LMICs (53–57).

Underlying mechanisms which may play an important role in HICs include delayed diagnosis in some social groups (123), communication barriers with health care professionals (124), differences in family's social resources, demands and health literacy (125) and parents' and child's adherence to medication and treatment recommendations (61, 124). Treatment adherence will have a greater effect upon outcome in malignancies such as ALL (61, 126) for which outpatient oral maintenance methotrexate/thiopurine therapy plays a major role and treatment usually last several years (127). A study from the UK revealed that socioeconomic differences in survival from ALL emerged about 8–9 months after the diagnosis (61) which is about the time when a child get typically discharged from hospital and continuation of therapy requires parental/child's adherence (including daily drug intake and frequent outpatient appointments). The authors hypothesized that this may be due to treatment adherence (61). Besides treatment adherence, second-line therapy might play an important role in survival difference in HIC. If first-line therapy fails, there are rarely well-established second-line therapies. The decision to treat with an intent to cure reflects the attitude and the resources of both the physicians and the family (128). SEP and in particular education may play a significant role here, since aggressive second-line therapy to obtain cure will reflect the family's understanding of the complexities and their ability to “co-decide” with the physician to initiate such therapies.

However, although survival inequalities exist in HICs, most children with poor socioeconomic SEP will still do substantially better than the children with cancer with higher SEP in resource-poor settings.

Family conditions are also found to be associated with survival. However, the evidence is limited to observations in HICs (58). Family factors are likely to play an important role as children rely on their parents help and support, as well as on the parents' ability to observe and communicate conditions of relevance for their child's health and treatment to the health care professionals. For some European populations (59, 60, 64, 69, 129, 130) the demands on families and their social resources appear to be more or equally relevant as compared to the socioeconomic situation of the family.

#### Socioeconomic Implications

In turn, social inequities may also be created or compounded by the childhood cancer diagnosis. The empirically observed socioeconomic implications on the parental situation are likely to be related to the demanding caregiving, emotional, and practical strains which parents with a sick child have to handle (131). The child's acute treatment requires frequent hospitalizations, invasive procedures, and depending on the cancer type, a combination of surgery, chemotherapy, or radiotherapy (132). Managing the child's disease and demanding treatment alongside family and work-related responsibilities is highly challenging. It may involve taking time off work, reducing or entirely leaving paid employment, with subsequent income reductions. Moreover, some parents reported elevated levels of distress even years after the child's acute treatment (133–135). The complex interplay of the parents' mental condition and socioeconomic implications has hardly been studied so far, but might also be an important underlying mechanism of adverse socioeconomic implications.

The empirically observed groups of survivors at particular risk of adverse socioeconomic outcomes—survivors of a central nervous system tumor, survivors treated with cranial radiotherapy and those diagnosed at younger age (90)—give some indication for the underlying mechanisms of the socioeconomic difficulties that some childhood cancer survivors face in their later life. The most obvious pathway is related to treatment and in particular radiation therapy received. Cranial irradiation has been associated with a large number of somatic late effects including long-term neurocognitive impairments, including fatigue, vision or hearing deficits as well as problems with concentration, learning and memory function (136–140). Young age at cancer treatment might affect the growing tissues and development with bones becoming deformed, tissue fibrosis development and organ functions being impaired, which may cause a large variety of morbidities and cognitive impairments (136, 140, 141) and also lead to educational and occupational difficulties.

However, socioeconomic difficulties in childhood cancer survivors might not only relate to the childhood cancer treatment itself, but also to the psychosocial stressors related to the disease and its demanding treatment. Survivors are at increased risk of mental conditions manifesting several years after the cancer diagnosis and treatment (142, 143). Moreover, the ability of the parents and care givers, the child with cancer and the entire families to handle a stressor like a cancer diagnosis and the availability of internal and external resistance resources, such as cognitive skills, management of emotional distress, and social support, may influence the socioeconomic situation of the childhood cancer survivors, also in a long-term perspective (90, 144, 145). Further, absence from school during the active treatment period may cause difficulties with reaching similar educational accomplishments as their peers and educational delays (146). How well a family is able to make up for absence from school might be related to the family social resources as well as to the parent's socioeconomic background. Somatic late effects and educational difficulties may limit the ability to work or to attain higher occupational positions, with subsequent lower opportunities to reach higher income levels and independence from social security benefits (90). Finally, some national legislation and policies might contribute to the socioeconomic difficulties of childhood cancer survivors. In some countries childhood survivors are not sufficiently protected from discrimination and finical disadvantages, including equal access to loans and insurances (147).

## Conclusion

The underlying mechanisms and pathways of the empirically demonstrated social inequalities along the childhood cancer continuum are poorly understood. We developed a conceptual framework specifically on social inequities in childhood cancer postulating mechanisms and pathways by which social determinants may create health inequalities and point out how major determinants relate to each other. Important identified and hypothesized underlying mechanisms and pathways of social inequity along the childhood cancer continuum which could be targets of future interventions and policy strategies to reduce childhood cancer-related inequity include timely and equal access to first-line diagnostics, contemporary therapy and supportive care, social support for families with a child with cancer, long-term follow-up of vulnerable groups of childhood cancer survivors to identify early signs of somatic and psychiatric late effects and adverse socioeconomic or psychosocial conditions, as well as a legal framework to protect cancer patients, survivors and their families from discrimination.

As an enhancement to previous conceptual frameworks, our framework takes the course of childhood cancer into account and stresses that each phase of the childhood cancer continuum is influenced by varying social determinants. Moreover, our framework distinguishes between social determinants of inequities in childhood cancer and social determinants of childhood cancer and thereby point toward the mechanisms by which social inequities are created and the determinants which impact directly on the childhood cancer continuum. The framework aims to be comprehensive and should be applicable to social inequities in childhood cancer on a global scale.

Although the proposed mechanisms and pathways are based on the best available scientific evidence, some are rather speculative than evidence-based. We understand this conceptual framework as work in progress which is thought to identify research gaps and help directing scientific research. Nevertheless, it may already be useful to identify potential targets for tailored approaches and better policy making, thus tackling social inequalities in childhood cancer and enhance equity.

## Author Contributions

FE and HZ developed the concept of the manuscript. FE conducted the literature search. FE and HZ developed the conceptual framework with input from MF, HM, and KS. FE drafted the manuscript. All authors provided critical feedback, revised the manuscript for intellectual content and approved the final version.

### Conflict of Interest Statement

The authors declare that the research was conducted in the absence of any commercial or financial relationships that could be construed as a potential conflict of interest. The reviewer JL-T declared a shared affiliation, with no collaboration within the past 5 years, with one of the authors, FE, to the handling editor at time of review.
